# Increased gene dosage and mRNA expression from chromosomal duplications in *Caenorhabditis elegans*

**DOI:** 10.1093/g3journal/jkac151

**Published:** 2022-06-22

**Authors:** Bhavana Ragipani, Sarah Elizabeth Albritton, Ana Karina Morao, Diogo Mesquita, Maxwell Kramer, Sevinç Ercan

**Affiliations:** Department of Biology, Center for Genomics and Systems Biology, New York University, New York, NY 10003, USA; Department of Biology, Center for Genomics and Systems Biology, New York University, New York, NY 10003, USA; Department of Biology, Center for Genomics and Systems Biology, New York University, New York, NY 10003, USA; Department of Biology, Center for Genomics and Systems Biology, New York University, New York, NY 10003, USA; Department of Biology, Center for Genomics and Systems Biology, New York University, New York, NY 10003, USA; Department of Biology, Center for Genomics and Systems Biology, New York University, New York, NY 10003, USA

**Keywords:** gene dosage, copy number, dosage compensation, X chromosome, RNA-seq, DNA-seq, chromosome duplications, *Caenorhabditis elegans*, modENCODE, gene expression

## Abstract

Isolation of copy number variations and chromosomal duplications at high frequency in the laboratory suggested that *Caenorhabditis elegans* tolerates increased gene dosage. Here, we addressed if a general dosage compensation mechanism acts at the level of mRNA expression in *C. elegans*. We characterized gene dosage and mRNA expression in 3 chromosomal duplications and a fosmid integration strain using DNA-seq and mRNA-seq. Our results show that on average, increased gene dosage leads to increased mRNA expression, pointing to a lack of genome-wide dosage compensation. Different genes within the same chromosomal duplication show variable levels of mRNA increase, suggesting feedback regulation of individual genes. Somatic dosage compensation and germline repression reduce the level of mRNA increase from X chromosomal duplications. Together, our results show a lack of genome-wide dosage compensation mechanism acting at the mRNA level in *C. elegans* and highlight the role of epigenetic and individual gene regulation contributing to the varied consequences of increased gene dosage.

## Introduction

Chromosomal aneuploidies or copy number variations (CNVs) are associated with a wide range of phenotypes in many organisms (Tang and Amon 2013; Durrbaum and Storchova 2016). To understand the effect of gene dosage on gene expression, a series of studies compared DNA copy number and mRNA levels in aneuploid cells and animals (Kojima and Cimini 2019). In plants and mammalian cells, partial and full aneuploidies showed complex responses to alterations in gene dosage, with secondary effects on the transcriptome and nonlinear correlation between copy number and expression (Aït Yahya-Graison *et al.* 2007; Huettel *et al.* 2008). In *Drosophila melanogaster*, buffering mechanisms were proposed to act through the gene regulatory networks to dampen the effect of gene dosage (Zhang *et al.* 2010; Malone *et al.* 2012). In *Saccharomyces cerevisiae*, gene expression largely correlated with copy number with some variation between genes (Gasch *et al.* 2016; Torres *et al.* 2016; Taggart and Li 2018).

While there is no effective compensation of chromosomal aneuploidies for the rest of the genome, X chromosomes are highly regulated in many animals. Typically, females (XX) contain twice the number of X chromosomes than males (XY) but the 2 sexes express similar levels of X chromosomal transcripts. Strategies that equalize X chromosome expression between sexes differ in different animals. In mammals (human and mouse), one of the X chromosomes is silenced in XX females; in flies (*Drosophila*), the single X chromosome is upregulated by a factor of 2 in XY males; and in worms (*Caenorhabditis*), both X chromosomes are downregulated by a factor of 2 in XX hermaphrodites (Meyer 2005; Samata and Akhtar 2018; Dossin and Heard 2021). While strategies differ, in each system, a multisubunit protein complex specifically targets and regulates X chromosome transcription in one of the 2 sexes through epigenetic mechanisms (Ercan 2015).

A high rate of CNVs was reported in mutation accumulation experiments in *Caenorhabditis elegans* (Lipinski *et al.* 2011; Konrad *et al.* 2018). In addition, 80% of the genome could be isolated as chromosomal duplications in the laboratory, leading to the conclusion that the worm is relatively tolerant to increased gene dosage (Hodgkin 2005). To understand the effect of increased gene dosage in *C. elegans*, we characterized large chromosomal duplications in several strains using DNA-seq and analyzed the effect of increased gene dosage in 3 chromosomal duplications and 1 fosmid insertion strain using mRNA-seq. Like other species, there was a complex response to increased gene dosage in *C. elegans*. While the average mRNA level increased for genes located within large duplications and the integrated fosmid, genes within the same chromosomal duplication showed varying levels of mRNA increase. An X chromosomal duplication that recruits the somatic dosage compensation complex (DCC) showed lower mRNA increase compared to one that did not recruit, also demonstrating the contribution of epigenetic regulation to the effect of gene dosage.

## Materials and methods

### Strains

Unless otherwise noted, strains were maintained at 20°C on nematode growth medium agar plates using standard *C. elegans* growth methods. TY1916 [yDp11 (X; IV); lon-2(e678) unc-9(e101) X] contains duplication (yDp11), producing long, non-Unc homozygous hermaphrodites. SP117 [mnDp10 (X; I); unc-3(e151) X] produces wild-type looking homozygous hermaphrodites. SP219 [mnDp1 (X; V)/+ V; unc-3(e151) X] contains the duplication (mnDp1), which is homozygous lethal, thus, wild-type looking heterozygous hermaphrodites containing the duplication were picked. BC4289 [sDp10 (IV; X)] contains the homozygous-viable duplication (sDP10). SP1981 [unc-115(mn481) dpy-6(e14) X; stDp2 (X; II)/+] contains homozygous-lethal duplication (stDP2). Wild-type looking heterozygous hermaphrodites were picked. VC100 [unc-112(r367) V; gkDf2 X] contains the homozygous-viable deletion (gkDf2) and produces wild-type looking hermaphrodites. OP37 {wgIs37 [pha-4::TY1::EGFP::3xFLAG + unc-119(+)]} is wild-type looking and generated by Sarov *et al.* (2012).

### DNA-seq

At least 20 worms were hand-picked as young adults. Worms were washed by settling animals at least 3 times with 1 ml M9 and starved overnight to remove gut bacteria. Following a final M9 wash, worms were resuspended in 100 µl TE and frozen. For DNA isolation, 400 μl of lysis buffer (0.1 M Tris–HCl; 0.1 M NaCl; 50 mM EDTA; 1.25% SDS) was added and worms were sonicated using Bioruptor 30 s on/off at high for 30 min. Sonicated DNA was isolated using Qiagen MinElute kit and Illumina DNA sequencing libraries were prepared as described previously (Albritton *et al.* 2014). Single- or paired-end sequencing was performed using Illumina HiSeq-2000 and aligned to genome version WS220 (ce10) using Bowtie2 (version 2.3.2) with default settings (Langmead and Salzberg 2012). All replicate and read number information is provided in Supplementary File 1. Samtools version 1.6 (Li 2011) was used to merge replicates before running bamCompare from Deeptools version 3.3.1 (Ramírez *et al.* 2016), using the following options: –binSize 500, –scaleFactorsMethod None, –normalizeUsing CPM, –operation log2, –minMappingQuality 30, –outFileFormat bedgraph, and –ignoreDuplicates. Copy number analysis was performed with CNVnator version 0.3.3 (Abyzov *et al.* 2011) comparing data from mutant strains to reference genome WS220 (ce10) using bin_size = 1,000. CNVnator output files listing deletions and duplications are provided in Supplementary File 2. Overlap of CNVs with genes was determined by Galaxy (https://usegalaxy.org/) tools using coverage option in “Operate on Genomic Intervals” (Afgan *et al.* 2018) and provided in Supplementary File 3. Only genes within the duplications and deletions greater than 10 kb were considered for further analysis.

### mRNA-seq

Mixed-stage embryos were collected from N2, TY1916, BC4289 and SP117 by bleaching gravid adults. L2-L3 worms were collected by plating embryos and growing for 22–26 h at 22.5°C, and young adults next day. Worms were resuspended in at least 10 volumes of Trizol and stored at −80°C. RNA preparation was performed as previously (Albritton *et al.* 2014). Briefly, samples were freeze cracked 3–5 times, followed by TRIzol purification, and cleaned up with Qiagen RNeasy kit. From 0.5 to 10 μg of total RNA, mRNA was purified using Sera-Mag oligo(dT) beads (Thermo Scientific), sheared and stranded Illumina sequencing libraries were prepared using a previously published protocol (Parkhomchuk *et al.* 2009). Sequencing was performed with Illumina HiSeq-2000 and reads were aligned to genome version WS220 with Hisat2 version 2.2.1 (Kim *et al.* 2019) using default parameters. Count data were calculated using HTSeq version 0.13.5 (Putri *et al.* 2022) and differential expression was performed using the R package DESeq2 version 1.30.1 (Love *et al.* 2014). FPKM values were generated using Cufflinks version 2.2.1 with options -p 8 –library type fr- first strand (Trapnell *et al.* 2010). FPKM and DEseq2 output are provided in Supplementary File 4. In Fig. 3, log2 fold change values were median centered by subtracting the genome median from each value.

### Gene enrichment analysis in OP37

Genes differentially expressed in OP37 compared to N2 were analyzed using the Wormbase tool Gene Set Enrichment Analysis (Angeles-Albores *et al.* 2016). PHA-4 ChIP-seq binding peaks from OP37 L3 larvae were downloaded from the modERN project (Kudron *et al.* 2018). The results of the enrichment analyses and the list of differentially expressed genes are provided in Supplementary File 5.

## Results

### Characterization of chromosomal duplications and deletions using DNA-seq

**Fig. 1.**
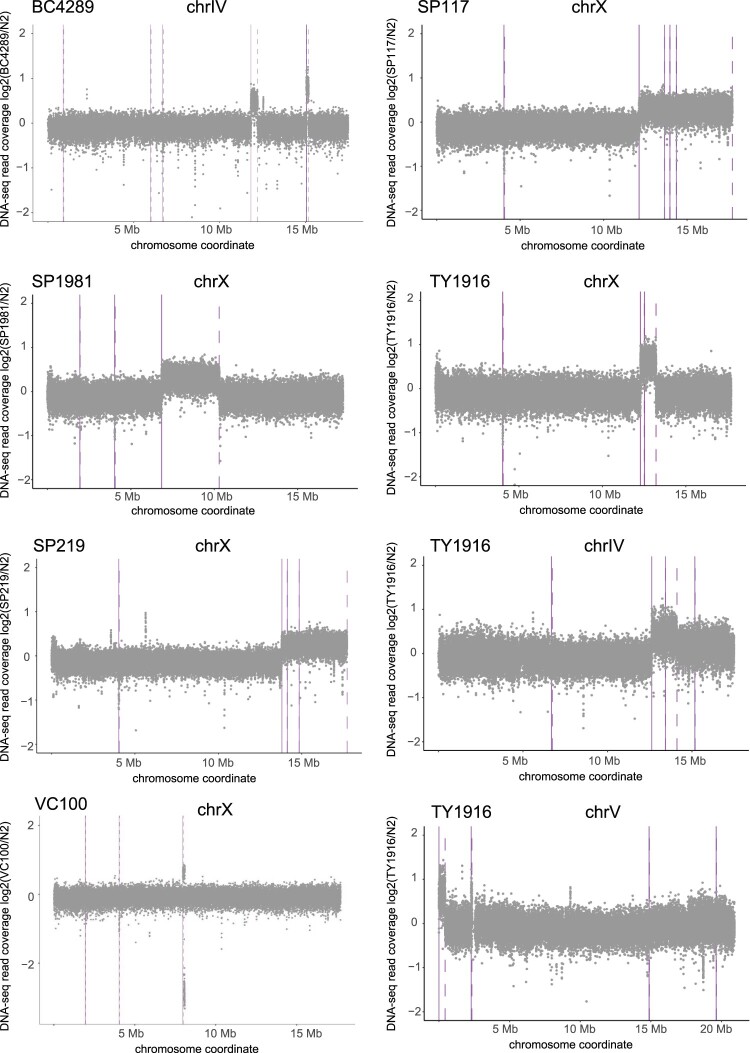
DNA-seq analysis of large chromosomal duplications. The *x*-axes are coordinates across the chromosomes containing duplications in each strain. The *y*-axis is log2(mutant/N2 control) coverage [CPM (counts per million) normalized and averaged for 1-kb windows]. Solid vertical lines indicate the start of a duplication and dotted lines indicate the end of a duplication as determined by CNVnator output.

To analyze the effect of gene dosage on mRNA expression, we used previously isolated strains with megabase-scale duplications (Supplementary File 1). Since prior characterization of the duplications was done by visible genetic markers (Hodgkin 2005; Edgley *et al.* 2006), we performed DNA-seq to map genes that were duplicated or deleted. First, we calculated average read coverage within 1-kb windows tiled across the genome. Plotting the ratio of coverage to wild type confirmed previously mapped duplications (Fig. 1).

**Fig. 2.**
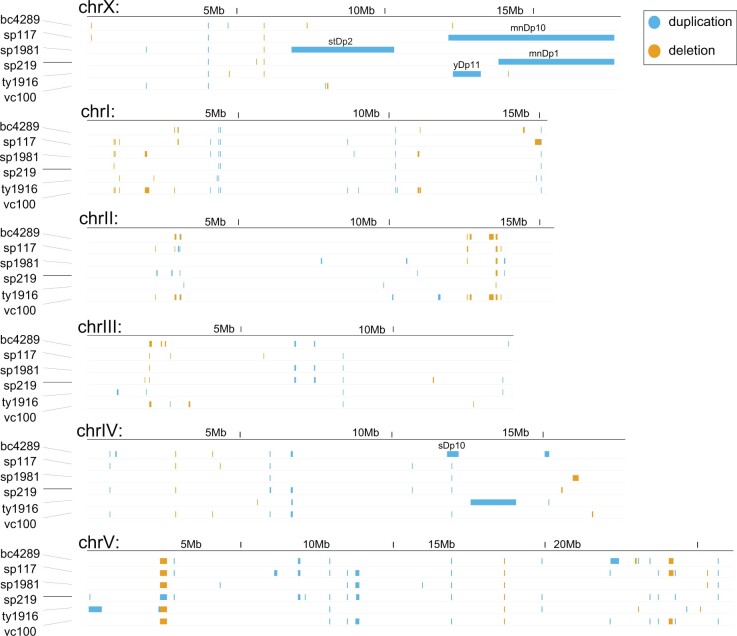
CNVnator-identified duplications and deletions. CNVnator identified duplications and deletions from each strain are visualized across the whole genome. Output files for each strain are provided in Supplementary File 2. Duplications are shown as blue, and deletions are shown as orange bars. The previously characterized large chromosomal duplications are labeled in each strain.

To further characterize the strains, we used CNVnator to generate a list of genomic windows with increased and decreased coverage compared to N2 (Abyzov *et al.* 2011; Supplementary File 2). In addition to the mapped changes, many of the strains showed smaller CNVs that were different from the N2 strain (Fig. 2). Some CNVs were common among the duplication strains, which may have originated from a laboratory strain polymorphic to N2 (Vergara *et al.* 2009). Notably, there were multiple smaller deletions and insertions within and at the boundaries of the larger duplications (Supplementary File 2). One example is the presence of a duplication near ∼40 kb deletion on the X chromosome in the VC100 strain. The presence of additional changes in the boundaries may be due to imperfect repair of the double-strand breaks used to induce aneuploidies, followed by selection to laboratory conditions (Farslow *et al.* 2015).

### Increased gene dosage and mRNA expression from chromosomal duplications

To analyze the effect of chromosomal duplications on gene expression, we performed mRNA-seq in 2 homozygous-viable strains with various length and number of duplications. SP117 contains a ∼5 Mb X chromosomal duplication attached to chromosome I (mnDp10). TY1916 contains a ∼1 Mb duplication from the X chromosome attached to chromosome IV (yDp11). TY1916 also contains previously uncharacterized changes involving the right arm of chromosome IV and the left end of chromosome V (Fig. 1). The left tip of chromosome V coverage (0.67) is similar to that of the characterized duplication from the X chromosome (0.60), thus appears to be duplicated. In the case of region with increased coverage on chromosome IV, the median ratio of coverage is lower (0.40) than that of the X duplication (0.60), thus this region may be present as free duplication or heterozygous in the population of worms used for DNA-seq.

**Fig. 3.**
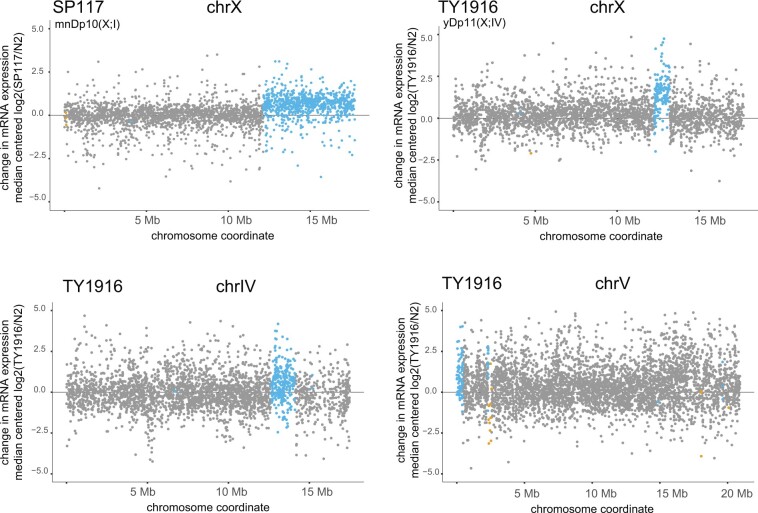
mRNA-seq analysis of 4 duplicated regions in 2 strains. Changes in mRNA expression in the SP117 and TY1916 strains compared to N2 are shown. In each graph, the *x*-axis shows the entire chromosomes from which the duplications originated from. The *y*-axis is the genome-median centered log2 ratio (mutant/N2) as determined by DESeq2. The DESeq2 outputs are provided in Supplementary File 4. The duplicated genes are highlighted in blue, deleted genes in orange, and unaffected genes in gray. Wilcoxon rank-sum test *P-*values are as follows: SP117 X duplicated versus X unaffected <2.2e−16, TY1916 X duplicated versus X unaffected <2.2e−16, TY1916 V-duplicated versus V-unaffected = 0.0016, TY1916 IV-duplicated versus IV-unaffected = 0.0029. Gene categories for all analyzed strains are provided in Supplementary File 3.

To identify the genes affected by the chromosomal duplications, we used the CNVnator defined regions and categorized genes as duplicated or deleted (if transcription start-end of the gene fully overlaps with the CNV), affected (if overlap is 1 bp or more), or unaffected (no overlap; Supplementary File 3). We then plotted the log2 ratio of mRNA coverage in the duplicated strain compared to control along the wild-type chromosomes, highlighting genes within each category. Across all duplications, average mRNA-seq expression was increased, suggesting that gene dosage correlates with mRNA expression in *C. elegans* (Fig. 3). Notably, the effect on individual genes was variable, showing a range of log2 ratios for genes located within the same duplication (Supplementary File 4).

In mRNA-seq analyses using ratios, inclusion of lowly expressed genes reduces the magnitude of observed effect (Deng *et al.* 2011). To address this problem, we filtered out genes with FPKM values less than 1 in any wild-type replicate, and replotted the 3 large duplications in SP117 and TY1916 (Supplementary Fig. 1a). The median mRNA-seq increase for all 3 duplications was similar with or without filtering (1.58 vs 1.51 for mnDp10, 2.34 vs 2.49 for yDp11, 1.51 vs 1.43 for duplication on chr IV in TY1916), thus lowly expressed genes did not significantly skew the analysis. We also plotted FPKM expression values for each gene between the large duplication strain SP117 and wild type (Supplementary Fig. 1b). Although the correlation is noisier at lower FPKM values, the shift in mRNA level is clear for genes located at the duplication.

To probe further into variability, we addressed if the tighter scatter of log2 ratios on the X chromosome (Fig. 3) is due to lower expression noise, which was shown for the single X chromosome upregulated by the DCC in flies (Lee *et al.* 2018). To address noise, we calculated the coefficient of variation for each gene (filtering out those with FPKM < 1) using wild-type mRNA-seq data replicates in embryos, larvae, and young adults (Supplementary Fig. 1c). Overall, there was less variation in larvae, likely due to temporal dynamics of embryogenesis in embryos and germ cell development in young adults adding variability between collection of worms for mRNA-seq replicates. In all 3 developmental stages, the median coefficient of variation for X chromosomal genes was in between other chromosomes, suggesting that dosage compensation in worms does not reduce expression noise below that of autosomes.

### Increased mRNA expression and indirect effects of a multicopy integrated fosmid

While megabase-scale chromosome duplications showed an average increase in mRNA level, we wondered if smaller chromosomal segments with higher copy numbers also increase mRNA expression. To this end, we used a multicopy fosmid integration strain, where GFP-3xflag tag was inserted to the C terminus of *pha-4* gene within a fosmid containing 3 other genes (Sarov *et al.* 2012). The fosmid was then integrated into the genome randomly and a transgenic line that was vigorous and expressing the GFP-tagged *pha-4* was selected. This strain named OP37 was used to study the binding sites of *pha-4*, a transcription factor required for several developmental processes including the pharynx (Gaudet and Mango 2002; Zhong *et al.* 2010). The copy number for the fosmid was calculated to be 5.6 (Sarov *et al.* 2012).

**Fig. 4.**
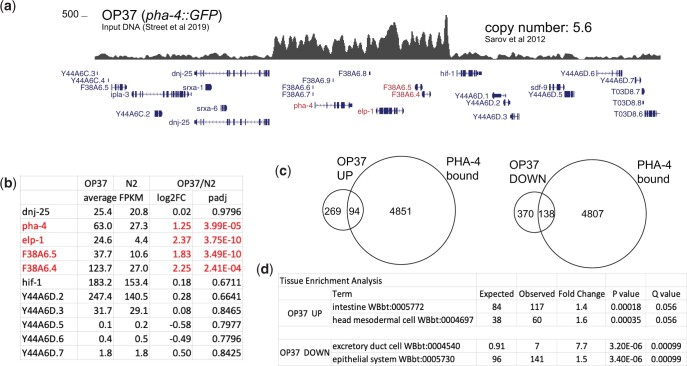
mRNA-seq analysis in a multicopy integrated fosmid strain. a) DNA-seq read coverage plot of OP37 visualized on UCSC genome browser, centered at a ∼70-kb window across the region where *pha-4* containing fosmid originated from. Genes included in the fosmid are highlighted in red. Only the longest isoforms are shown for simplicity. b) Table showing the average FPKM, log2 fold change, and *P*adj values from DESeq2 analyses, listed for genes within and neighboring the *pha-4* containing fosmid. The genes within the fosmid are highlighted in red. The entire analysis is included in Supplementary File 4. c) Overlap of genes bound by PHA-4 (modENCODE ChIP-seq peaks at 1-kb promoters) and genes up- or downregulated in the OP37 strain compared to N2. d) Tissue enrichment analysis of differentially expressed genes in the OP37 strain. Top 2 categories are shown for up- and downregulated genes, and the rest are provided in Supplementary File 5.

The genes within and surrounding where the fosmid originated from are shown along with the DNA read coverage highlighting the increased gene dosage (Fig. 4a). The insertion site of the fosmid has not been mapped. Notably, the mRNA level of all 4 genes including *pha-4* was increased in the OP37 strain compared to N2 control (Fig. 4b). The increase for *pha-4* was by a factor of ∼2.4 suggesting either feedback repression or an effect of GFP tagging on mRNA expression or detection. The mRNA level of the other 3 genes on the fosmid increased by a factor of ∼3.6- to 4.8, reflecting the increase in their copy number.

Highlighting the indirect effect on the transcriptome, 871 genes were identified to be up- or downregulated in the OP37 strain, despite the fosmid including only 4 genes. Analysis of genes that were differentially expressed revealed no enrichment for PHA-4 bound genes (25% of upregulated and 27% of downregulated genes were bound by PHA-4 compared to 27% of all genes; Fig. 4c). It is possible that ∼2.4-fold increase in *pha-4* mRNA level does not cause an increase at the protein level or that the presence of multiple genes within the fosmid reduces *pha-4* specific changes on the transcriptome. The differentially expressed genes in the OP37 strain showed tissue-specific enrichment possibly reflecting the developmental functions of *pha-4* and the neighboring genes (Fig. 4d; Supplementary File 5).

### The mRNA increase from a X duplication differs across developmental stages

**Fig. 5.**
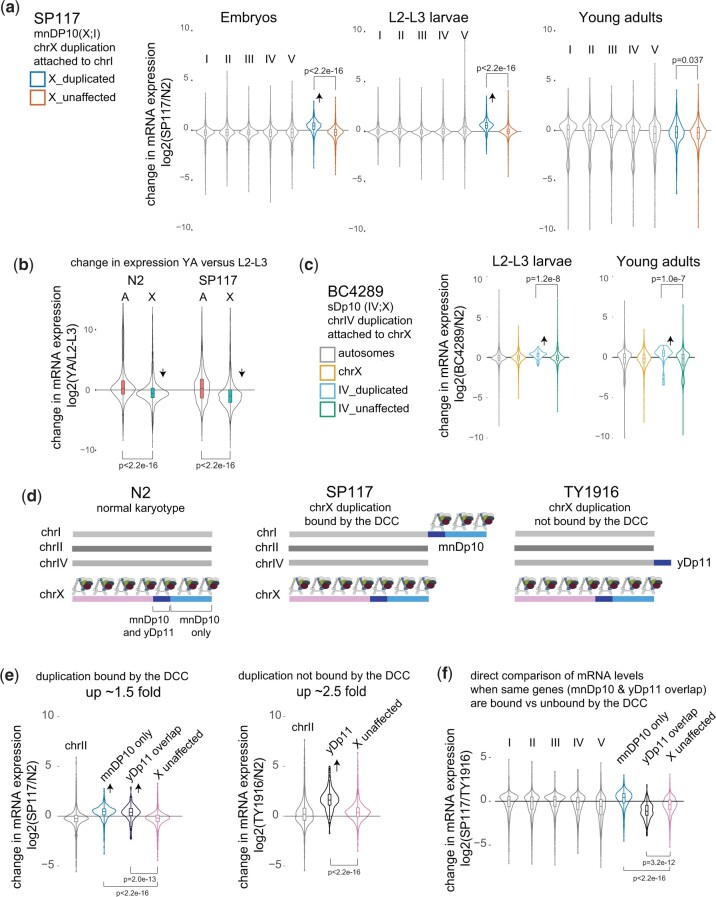
mRNA expression in X duplications subject to repression in the soma and germline. a) SP117 contains a large ∼5 Mb duplication of chr X that is attached to chr I. Violin plots of log2 fold change from DESeq2 analysis of SP117 compared to N2 in embryos, L2-L3 larvae and young adult worms. Significance of difference between average log2 fold change for genes within and outside the duplication from chr X is tested by *t*-test. Increased mRNA expression from the duplication in embryos and larvae is highlighted with an arrow. b) Violin plot of log2 fold change from DESeq2 analysis of YA to L2-L3 in N2 and SP117. X chromosomes are repressed in the germ cells that are present in young adults but not in L2-L3 larvae Difference in average change in expression between X and A is tested by *t*-test. c) BC4289 contains a ∼1 Mb chr IV duplication attached to chr X. Violin plots of log2 fold change from DESeq2 analysis of BC4289 compared to N2 in L2-L3 larvae and young adults is shown. Significance of difference between average log2 fold change of genes within and outside the duplication from chr IV is tested using *t*-test. d) A schematic representing N2 control, SP117 and TY1916 genomes. The 1 Mb duplicated region (dark blue) in TY1916 (yDp11) does not bind to the somatic DCC. The 4 Mb uniquely duplicated region in SP117 (mnDp10) is indicated in light blue. Within mnDp10, genes within the duplicated region that overlap with yDp11 (dark blue) is bound by the DCC. e) Violin plots of log2 fold change of SP117 and TY1916 compared to N2 in L2-L3 larvae. The 1.5- and 2.5-fold refer to the ratio of the median for the 1 Mb common duplicated region compared to the unaffected X chromosomal genes [lowly expressed genes are filtered (FPKM < 1)]. The arrows indicate the mRNA-seq increase in the duplicated regions, with *P*-values generated from *t*-test. f) Violin plot of log2 fold change ratio for SP117 directly compared to TY1916. The increase in mRNA expression of the same set of genes is lower in SP117 when ∼1 Mb X chromosome duplication is bound by the DCC compared to when unbound in TY1916.

Next, we wondered how epigenetic regulation of the X chromosomes in different tissues would affect the consequence of an X chromosomal duplication. To address this question, we first considered the germline, where the X chromosomal genes are repressed compared to autosomal genes during early meiosis (Schaner and Kelly 2006). We reasoned that if a X chromosomal duplication is also subject to repression, the mRNA increase would be lower in adult worms. Indeed, genes located within the 5 Mb X chromosomal duplication in the SP117 strain showed less increase in mRNA expression in young adults compared to embryo and larvae (Fig. 5a). X chromosome repression in the germ cells is measurable in whole animals because germ cells outnumber the somatic cells in adults, leading to lower X chromosome expression compared to autosomes (Fig. 5b). Thus, our results suggest that repression of genes within the 5 Mb X chromosomal duplication in the germline negates the mRNA effect of the duplications specifically in this tissue.

The duplication strain BC4289 contains a ∼1 Mb translocation from chromosome IV to X allowing us to test if autosomal genes attached to the X chromosome are repressed in germ cells. Unlike the 5 Mb X-to-I translocation in SP117, the 1 Mb IV-to-X translocation in BC4289 showed a similar increase in mRNA expression in young adults compared to larvae (Fig. 5c). Thus, germline repression may not affect autosomal genes even when they are physically attached to the X chromosome. In the future, multiple autosome to X duplications should be analyzed to test the generality of this conclusion. Nevertheless, the SP117 data suggest that epigenetic regulation of X chromosomal genes reduces the effect of a large X duplication on mRNA expression in the germline.

### Somatic dosage compensation reduces the level of mRNA increase in X chromosomal duplications

In *C. elegans*, hermaphrodite X chromosomes are repressed by a factor of 2 by somatic dosage compensation (Kramer *et al.* 2016). This repression is mediated by a condensin-based DCC that specifically binds to and represses transcription initiation from both X chromosomes (Kruesi *et al.* 2013; Kramer *et al.* 2015). DCC is recruited specifically to the X chromosomes by several cis-regulatory elements on the X (McDonel *et al.* 2006; Ercan *et al.* 2007; Jans *et al.* 2009; Albritton and Ercan 2017). Recruitment leads to spreading of the complex to nearby chromatin, creating robust binding across the X chromosomes (Albritton *et al.* 2017; Street *et al.* 2019).

Previous studies used immunofluorescence to analyze the ability of several X chromosomal duplications to recruit the DCC (Csankovszki *et al.* 2004; Blauwkamp and Csankovszki 2009). These studies found that ∼1 Mb duplication in TY1916 (yDp11) does not recruit the DCC. Although one strong and one weak recruitment sites are present within yDp11, insertion of few recruitment elements is not sufficient to recruit high levels of DCC to an autosome (Albritton *et al.* 2017), explaining the lack of robust DCC binding to this shorter duplication. The same stretch of DNA within the larger ∼5 Mb duplication in SP117 (mnDP10), is bound by the DCC (Csankovszki *et al.* 2004), due to the additional ∼4 Mb region containing 5 strong and 15 weaker recruitment elements. Since genes in yDp11 are also in mnDP10, we were able to test if epigenetic repression by the DCC reduced the effect of increased dosage across the shared genes (Fig. 5d). Indeed, the average mRNA increase from the 1 Mb region differed in the 2 strains (Fig. 5e). The median increase of mRNA expression from the 1 Mb duplication was lower when bound by the DCC (SP117) compared to when unbound (TY1916).

In TY1916, there are 4 copies of genes located within the 1 Mb yDp11; 2 X chromosomal copies under DCC-mediated repression and 2 duplicated copies on chromosome IV without DCC. In SP117, all 4 copies of the genes within the commonly duplicated region between mnDp10 and yDP11 are under DCC-mediated repression. In both strains, the expected level of median mRNA increase (based on copy number and the assumed DCC repression by a factor of 2) was lower than that of observed; ∼2.3-fold vs 3 in TY1916 and ∼1.5-fold vs 2 in SP117 [after filtering genes (FPKM < 1) median increase was ∼2.5 for TY1916 and 1.5 for SP117]. It would be interesting to know if other mechanisms of repression such as lamina-mediated organization of repressive chromatin contribute to repression of X chromosomal duplications (Snyder *et al.* 2016; Lee and Oliver 2018). Regardless, the lower level of mRNA increase from the same set of duplicated genes in the SP117 (DCC-bound) strain compared to TY1916 (DCC-unbound; Fig. 5f) indicates that DCC-mediated repression reduced the consequence of increased gene dosage on mRNA expression.

## Discussion

In *C. elegans*, several observations supported the idea that this organism is robust to changes in gene dosage in laboratory (Hodgkin 2005; Lipinski *et al.* 2011; Sarov *et al.* 2012; Konrad *et al.* 2018). Here, we showed that tolerance to increased gene dosage is not due to a genome-wide compensation mechanism acting at the mRNA level. However, it remains possible that effective mechanisms of compensation exist at the protein level (Dalley *et al.* 1993; Chang and Liao 2020). Post-transcriptional control of gene expression is common in *C. elegans* particularly in the germline (Nousch and Eckmann 2013), a tissue whose function is central to the viability of all isolated strains.

Our analysis of chromosomal duplications in *C. elegans* indicates that similar to other organisms (Kojima and Cimini 2019), increased gene dosage leads to increased mRNA expression with a high degree of variability between genes (Fig. 3). The variation in response to gene dosage could be due to measurement errors based on technical and biological variation in mRNA expression. It could also be due to feedback through gene regulatory networks (Malone *et al.* 2012). Indirect effect of increased gene dosage on the transcriptome was particularly evident in the fosmid integration strain (Fig. 4). It is possible that compensatory changes in the expression of other genes render chromosomal duplications and CNVs viable in the laboratory.

Partial aneuploidies and CNVs in specific genes have been implicated in a wide range of diseases (Inaki and Liu 2012; Tang and Amon 2013; Durrbaum and Storchova 2016). To determine the cause and consequences of these CNVs on organism phenotypes, it is necessary to understand the variation both between genes and between tissues. Here, our results demonstrate that tissue-specific mechanisms of X chromosomal gene repression control the level of mRNA increase from certain duplications in the germline and the soma. Thus, increased gene dosage could result in varying levels of mRNA-seq increase, when the same set of genes is under different epigenetic regulation. Overall, our work adds *C. elegans* to the line of research analyzing partial aneuploidies, demonstrating a lack of genome-wide dosage compensation mechanism acting at the mRNA level, highlighting gene-to-gene variability and the contribution of epigenetic regulation to the mRNA response to gene dosage.

## Data availability

A list of new and published data used in this study is provided in Supplementary File 1 with GEO accession numbers. The new data are available at GEO Series number GSE198682. Supplementary Material is included at figshare: https://doi.org/10.25387/g3.19762756.

## References

[jkac151-B1] Abyzov A , Urban AE, Snyder M, Gerstein M. CNVnator: an approach to discover, genotype, and characterize typical and atypical CNVs from family and population genome sequencing. Genome Res. 2011;21(6):974–984. 21324876 10.1101/gr.114876.110 PMC3106330

[jkac151-B2] Afgan E , Baker D, Batut B, van den Beek M, Bouvier D, Cech M, Chilton J, Clements D, Coraor N, Grüning BA, et al The Galaxy platform for accessible, reproducible and collaborative biomedical analyses: 2018 update. Nucleic Acids Res. 2018;46(W1):W537–W544. 29790989 10.1093/nar/gky379 PMC6030816

[jkac151-B3] Aït Yahya-Graison E , Aubert J, Dauphinot L, Rivals I, Prieur M, Golfier G, Rossier J, Personnaz L, Creau N, Bléhaut H, et al Classification of human chromosome 21 gene-expression variations in Down syndrome: impact on disease phenotypes. Am J Hum Genet. 2007;81(3):475–491. 17701894 10.1086/520000 PMC1950826

[jkac151-B4] Albritton SE , Ercan S. *Caenorhabditis elegans* dosage compensation: insights into condensin-mediated gene regulation. Trends Genet. 2017;34(1):41–53. 29037439 10.1016/j.tig.2017.09.010 PMC5741500

[jkac151-B5] Albritton SE , Kranz A-L, Rao P, Kramer M, Dieterich C, Ercan S. Sex-biased gene expression and evolution of the x chromosome in nematodes. Genetics. 2014;197(3):865–883. 24793291 10.1534/genetics.114.163311 PMC4096367

[jkac151-B6] Albritton SE , Kranz AL, Winterkorn LH, Street LA, Ercan S. Cooperation between a hierarchical set of recruitment sites targets the X chromosome for dosage compensation. Elife. 2017;6:e23645. 10.7554/eLife.23645 PMC5451215 28562241

[jkac151-B7] Angeles-Albores D , Lee RYN, Chan J, Sternberg PW. Tissue enrichment analysis for *C. elegans* genomics. BMC Bioinformatics. 2016;17(1):366. 27618863 10.1186/s12859-016-1229-9 PMC5020436

[jkac151-B8] Blauwkamp TA , Csankovszki G. Two classes of dosage compensation complex binding elements along *Caenorhabditis elegans* X chromosomes. Mol Cell Biol. 2009;29(8):2023–2031. 19188444 10.1128/MCB.01448-08 PMC2663307

[jkac151-B9] Chang AY , Liao BY. Reduced translational efficiency of eukaryotic genes after duplication events. Mol Biol Evol. 2020;37(5):1452–1461. 31904835 10.1093/molbev/msz309

[jkac151-B10] Csankovszki G , McDonel P, Meyer BJ. Recruitment and spreading of the *C. elegans* dosage compensation complex along X chromosomes. Science. 2004;303(5661):1182–1185. 14976312 10.1126/science.1092938

[jkac151-B11] Dalley BK , Rogalski TM, Tullis GE, Riddle DL, Golomb M. Post-transcriptional regulation of RNA polymerase II levels in *Caenorhabditis elegans*. Genetics. 1993;133(2):237–245. 8436272 10.1093/genetics/133.2.237 PMC1205314

[jkac151-B12] Deng X , Hiatt JB, Nguyen DK, Ercan S, Sturgill D, Hillier LW, Schlesinger F, Davis CA, Reinke VJ, Gingeras TR, et al Evidence for compensatory upregulation of expressed X-linked genes in mammals, *Caenorhabditis elegans* and *Drosophila melanogaster*. Nat Genet. 2011;43(12):1179–1185. 22019781 10.1038/ng.948 PMC3576853

[jkac151-B13] Dossin F , Heard E. The molecular and nuclear dynamics of X-chromosome inactivation. Cold Spring Harb Perspect Biol. 2021;14(4):a040196. 10.1101/cshperspect.a040196 PMC9121902 34312245

[jkac151-B14] Durrbaum M , Storchova Z. Effects of aneuploidy on gene expression: implications for cancer. FEBS J. 2016;283:791–802. 26555863 10.1111/febs.13591

[jkac151-B15] Edgley ML , Baillie DL, Riddle DL, Rose AM. Genetic balancers. In: WormBook. The *C. elegans* Research Community, editor. 2006. p. 1–32. 10.1895/wormbook.1.89.1 PMC4781404 18050450

[jkac151-B16] Ercan S. Mechanisms of x chromosome dosage compensation. J Genomics. 2015;3:1–19. 25628761 10.7150/jgen.10404 PMC4303597

[jkac151-B17] Ercan S , Giresi PG, Whittle CM, Zhang X, Green RD, Lieb JD. X chromosome repression by localization of the *C. elegans* dosage compensation machinery to sites of transcription initiation. Nat Genet. 2007;39(3):403–408. 17293863 10.1038/ng1983 PMC2753834

[jkac151-B18] Farslow JC , Lipinski KJ, Packard LB, Edgley ML, Taylor J, Flibotte S, Moerman DG, Katju V, Bergthorsson U. Rapid increase in frequency of gene copy-number variants during experimental evolution in *Caenorhabditis elegans*. BMC Genomics. 2015;16:1044. 26645535 10.1186/s12864-015-2253-2 PMC4673709

[jkac151-B19] Gasch AP , Hose J, Newton MA, Sardi M, Yong M, Wang Z. Further support for aneuploidy tolerance in wild yeast and effects of dosage compensation on gene copy-number evolution. Elife. 2016;5:e14409. 26949252 10.7554/eLife.14409 PMC4798956

[jkac151-B20] Gaudet J , Mango SE. Regulation of organogenesis by the *Caenorhabditis elegans* FoxA protein PHA-4. Science. 2002;295(5556):821–825. 11823633 10.1126/science.1065175

[jkac151-B21] Hodgkin J. Karyotype, ploidy, and gene dosage. In: WormBook. The *C. elegans* Research Community, editor. 2005. p. 1–9. 10.1895/wormbook.1.3.1 PMC4781021 18023124

[jkac151-B22] Huettel B , Kreil DP, Matzke M, Matzke AJ. Effects of aneuploidy on genome structure, expression, and interphase organization in *Arabidopsis thaliana*. PLoS Genet. 2008;4(10):e1000226. 18927630 10.1371/journal.pgen.1000226 PMC2562519

[jkac151-B23] Inaki K , Liu ET. Structural mutations in cancer: mechanistic and functional insights. Trends Genet. 2012;28(11):550–559. 22901976 10.1016/j.tig.2012.07.002

[jkac151-B24] Jans J , Gladden JM, Ralston EJ, Pickle CS, Michel AH, Pferdehirt RR, Eisen MB, Meyer BJ. A condensin-like dosage compensation complex acts at a distance to control expression throughout the genome. Genes Dev. 2009;23(5):602–618. 19270160 10.1101/gad.1751109 PMC2658519

[jkac151-B25] Kim D , Paggi JM, Park C, Bennett C, Salzberg SL. Graph-based genome alignment and genotyping with HISAT2 and HISAT-genotype. Nat Biotechnol. 2019;37(8):907–915. 31375807 10.1038/s41587-019-0201-4 PMC7605509

[jkac151-B26] Kojima S , Cimini D. Aneuploidy and gene expression: is there dosage compensation? Epigenomics. 2019;11(16):1827–1837. 31755744 10.2217/epi-2019-0135 PMC7132608

[jkac151-B27] Konrad A , Flibotte S, Taylor J, Waterston RH, Moerman DG, Bergthorsson U, Katju V. Mutational and transcriptional landscape of spontaneous gene duplications and deletions in *Caenorhabditis elegans*. Proc Natl Acad Sci U S A. 2018;115(28):7386–7391. 29941601 10.1073/pnas.1801930115 PMC6048555

[jkac151-B28] Kramer M , Kranz A-L, Su A, Winterkorn LH, Albritton SE, Ercan S. Developmental dynamics of X-chromosome dosage compensation by the DCC and H4K20me1 in *C. elegans*. PLoS Genet. 2015;11(12):e1005698. 26641248 10.1371/journal.pgen.1005698 PMC4671695

[jkac151-B29] Kramer M , Rao P, Ercan S. Untangling the contributions of sex-specific gene regulation and X-chromosome dosage to sex-biased gene expression in *Caenorhabditis elegans*. Genetics. 2016;204(1):355–369. 27356611 10.1534/genetics.116.190298 PMC5012400

[jkac151-B30] Kruesi WS , Core LJ, Waters CT, Lis JT, Meyer BJ. Condensin controls recruitment of RNA polymerase II to achieve nematode X-chromosome dosage compensation. Elife. 2013;2:e00808. 23795297 10.7554/eLife.00808 PMC3687364

[jkac151-B31] Kudron MM , Victorsen A, Gevirtzman L, Hillier LW, Fisher WW, Vafeados D, Kirkey M, Hammonds AS, Gersch J, Ammouri H, et al The ModERN resource: genome-wide binding profiles for hundreds of Drosophila and *Caenorhabditis elegans* transcription factors. Genetics. 2018;208(3):937–949. 29284660 10.1534/genetics.117.300657 PMC5844342

[jkac151-B32] Langmead B , Salzberg SL. Fast gapped-read alignment with Bowtie 2. Nat Methods. 2012;9(4):357–359. 22388286 10.1038/nmeth.1923 PMC3322381

[jkac151-B33] Lee H , Cho D-Y, Wojtowicz D, Harbison ST, Russell S, Oliver B, Przytycka TM. Dosage-dependent expression variation suppressed on the Drosophila male X chromosome. G3 (Bethesda). 2018;8(2):587–598. 29242386 10.1534/g3.117.300400 PMC5919722

[jkac151-B34] Lee H , Oliver B. Non-canonical Drosophila X chromosome dosage compensation and repressive topologically associated domains. Epigenetics Chromatin. 2018;11(1):62. 30355339 10.1186/s13072-018-0232-y PMC6199721

[jkac151-B35] Li H. A statistical framework for SNP calling, mutation discovery, association mapping and population genetical parameter estimation from sequencing data. Bioinformatics. 2011;27(21):2987–2993. 21903627 10.1093/bioinformatics/btr509 PMC3198575

[jkac151-B36] Lipinski KJ , Farslow JC, Fitzpatrick KA, Lynch M, Katju V, Bergthorsson U. High spontaneous rate of gene duplication in *Caenorhabditis elegans*. Curr Biol. 2011;21(4):306–310. 21295484 10.1016/j.cub.2011.01.026 PMC3056611

[jkac151-B37] Love MI , Huber W, Anders S. Moderated estimation of fold change and dispersion for RNA-seq data with DESeq2. Genome Biol. 2014;15(12):550. 25516281 10.1186/s13059-014-0550-8 PMC4302049

[jkac151-B38] Malone JH , Cho D-Y, Mattiuzzo NR, Artieri CG, Jiang L, Dale RK, Smith HE, McDaniel J, Munro S, Salit M, et al Mediation of Drosophila autosomal dosage effects and compensation by network interactions. Genome Biol. 2012;13(4):r28. 22531030 10.1186/gb-2012-13-4-r28 PMC3446302

[jkac151-B39] McDonel P , Jans J, Peterson BK, Meyer BJ. Clustered DNA motifs mark X chromosomes for repression by a dosage compensation complex. Nature. 2006;444(7119):614–618. 17122774 10.1038/nature05338 PMC2693371

[jkac151-B40] Meyer BJ. X-Chromosome dosage compensation. In: WormBook. The *C. elegans* Research Community, editor. 2005. p. 1–14. 10.1895/wormbook.1.8.1 PMC4781388 18050416

[jkac151-B41] Nousch M , Eckmann CR. Translational control in the *Caenorhabditis elegans* germ line. Adv Exp Med Biol. 2013;757:205–247. 22872479 10.1007/978-1-4614-4015-4_8

[jkac151-B42] Parkhomchuk D , Borodina T, Amstislavskiy V, Banaru M, Hallen L, Krobitsch S, Lehrach H, Soldatov A. Transcriptome analysis by strand-specific sequencing of complementary DNA. Nucleic Acids Res. 2009;37(18):e123. 19620212 10.1093/nar/gkp596 PMC2764448

[jkac151-B43] Putri GH , Anders S, Pyl PT, Pimanda JE, Zanini F. Analysing high-throughput sequencing data in Python with HTSeq 2.0. Bioinformatics. 2022;38(10):2943–2945. 35561197 10.1093/bioinformatics/btac166 PMC9113351

[jkac151-B44] Ramírez F , Ryan DP, Grüning B, Bhardwaj V, Kilpert F, Richter AS, Heyne S, Dündar F, Manke T. deepTools2: a next generation web server for deep-sequencing data analysis. Nucleic Acids Res. 2016;44(W1):W160–W165. 27079975 10.1093/nar/gkw257 PMC4987876

[jkac151-B45] Samata M , Akhtar A. Dosage compensation of the X chromosome: a complex epigenetic assignment involving chromatin regulators and long noncoding RNAs. Annu Rev Biochem. 2018;87:323–350. 29668306 10.1146/annurev-biochem-062917-011816

[jkac151-B46] Sarov M , Murray JI, Schanze K, Pozniakovski A, Niu W, Angermann K, Hasse S, Rupprecht M, Vinis E, Tinney M, et al A genome-scale resource for in vivo tag-based protein function exploration in *C. elegans*. Cell. 2012;150(4):855–866. 22901814 10.1016/j.cell.2012.08.001 PMC3979301

[jkac151-B47] Schaner CE , Kelly WG. Germline chromatin. In: WormBook. The *C. elegans* Research Community, editor. 2006. p. 1–14. 10.1895/wormbook.1.73.1 PMC4781259 18050477

[jkac151-B48] Snyder MJ , Lau AC, Brouhard EA, Davis MB, Jiang J, Sifuentes MH, Csankovszki G. Anchoring of heterochromatin to the nuclear lamina reinforces dosage compensation-mediated gene repression. PLoS Genet. 2016;12(9):e1006341. 27690361 10.1371/journal.pgen.1006341 PMC5045178

[jkac151-B49] Street LA , Morao AK, Winterkorn LH, Jiao C-Y, Albritton SE, Sadic M, Kramer M, Ercan S. Binding of an X-specific condensin correlates with a reduction in active histone modifications at gene regulatory elements. Genetics. 2019;212(3):729–742. 31123040 10.1534/genetics.119.302254 PMC6614895

[jkac151-B50] Taggart JC , Li GW. Production of protein-complex components is stoichiometric and lacks general feedback regulation in eukaryotes. Cell Syst. 2018;7(6):580–589.e4. 30553725 10.1016/j.cels.2018.11.003 PMC6659121

[jkac151-B51] Tang YC , Amon A. Gene copy-number alterations: a cost-benefit analysis. Cell. 2013;152(3):394–405. 23374337 10.1016/j.cell.2012.11.043 PMC3641674

[jkac151-B52] Torres EM , Springer M, Amon A. No current evidence for widespread dosage compensation in *S. cerevisiae*. Elife. 2016;5:e10996. 26949255 10.7554/eLife.10996 PMC4798953

[jkac151-B53] Trapnell C , Williams BA, Pertea G, Mortazavi A, Kwan G, van Baren MJ, Salzberg SL, Wold BJ, Pachter L. Transcript assembly and quantification by RNA-Seq reveals unannotated transcripts and isoform switching during cell differentiation. Nat Biotechnol. 2010;28(5):511–515. 20436464 10.1038/nbt.1621 PMC3146043

[jkac151-B54] Vergara IA , Mah AK, Huang JC, Tarailo-Graovac M, Johnsen RC, Baillie DL, Chen N. Polymorphic segmental duplication in the nematode *Caenorhabditis elegans*. BMC Genomics. 2009;10:329. 19622155 10.1186/1471-2164-10-329 PMC2728738

[jkac151-B55] Zhang Y , Malone JH, Powell SK, Periwal V, Spana E, Macalpine DM, Oliver B. Expression in aneuploid Drosophila S2 cells. PLoS Biol. 2010;8(2):e1000320. 20186269 10.1371/journal.pbio.1000320 PMC2826376

[jkac151-B56] Zhong M , Niu W, Lu ZJ, Sarov M, Murray JI, Janette J, Raha D, Sheaffer KL, Lam HYK, Preston E, et al Genome-wide identification of binding sites defines distinct functions for *Caenorhabditis elegans* PHA-4/FOXA in development and environmental response. PLoS Genet. 2010;6(2):e1000848. 20174564 10.1371/journal.pgen.1000848 PMC2824807

